# Histological Changes in the Cat Placenta Throughout Gestation

**DOI:** 10.3390/vetsci12030207

**Published:** 2025-03-01

**Authors:** Gimena Gomez Castro, Rocío Hernández, Andrea Cristofolini, Enrique Portiansky, Cecilia Merkis, Erika Badura, Luciano Casas, Claudio Barbeito, Mónica Diessler

**Affiliations:** 1 Laboratorio de Histología y Embriología Descriptiva, Experimental y Comparada (LHYEDEC), Facultad de Ciencias Veterinarias, Universidad Nacional de La Plata (FCV, UNLP), La Plata 1900, Argentina; rhernandez@fcv.unlp.edu.ar (R.H.); diessler@fcv.unlp.edu.ar (M.D.); 2 Consejo Nacional de Investigaciones Científicas y Técnicas (CONICET), CABA C1425FQB, La Plata 1900, Argentina; acristofolini@ayv.unrc.edu.ar (A.C.); elporti@fcv.unlp.edu.ar (E.P.); 3 Área de Microscopía Electrónica, Departamento de Patología Animal, Facultad de Agronomía y Veterinaria, Universidad Nacional de Río Cuarto, Río Cuarto 5800, Argentina; cmerkis@ayv.unrc.edu.ar; 4 Laboratorio de Análisis de Imágenes (LAI), FCV, UNLP, La Plata 1900, Argentina; 5 Cátedra de Enfermedades de Aves y Pilíferos, FCV, UNLP, La Plata 1900, Argentina; erikabadura@hotmail.com; 6 Servicio de Atención Clínica Primaria de Pequeños Animales, Hospital Escuela, FCV, UNLP, La Plata 1900, Argentina; casasluciano@fcv.unlp.edu.ar

**Keywords:** endotheliochorial placenta, feline, comparative placentology, trophoblast

## Abstract

This study on cat placental organogenesis provides a detailed histological description, emphasizing the stages that have been less described. Thirty-seven gestational sacs were obtained by ovariohysterectomy, and the gestational age of the embryos/fetuses was determined based on developmental characteristics. The placentas were measured and processed by routine histological techniques. Additionally, fresh tissue from a term placenta was processed for ultrastructural analysis. An in-depth histological analysis was performed, and several morphometric variables (placental and lamellar width, placental and labyrinthine thickness, area and number of decidual cells) were recorded and related mainly to gestational age. A significant increase was observed in fetal length from 31 dpc, while placental thickness rose until 39 dpc; lamellae became abundant, parallel, longer, and narrower. Many CTB cells gradually fused into the STB; however, it progressively reduced. Medium-sized decidual cells, arranged in groups at the junctional zone, were progressively incorporated into the lamellae; there, they persisted until term, decreasing in number and becoming larger and frequently binucleated. The description of temporal modifications in lamellar, trophoblastic, and decidual features widens current knowledge on feline placental morphogenesis. In addition, these findings might be valuable for elucidating mechanisms behind placental development, which in turn affect its efficiency.

## Simple Summary

The placenta is the organ that allows nutrition and development of the kitten fetuses inside the cat’s womb; it is formed by mother and embryo tissues. This study explores how the components of the cat placenta develop throughout gestation (as it has been previously described only at specific times). Gestational age was determined based on different features, including the length of embryos/fetuses (which increased significantly in the second half of pregnancy), the shape of digits, appearance of hair, hardening of claws, etc. Placental thickness increased until 39 days of gestation. Microscopic analysis of the placentas allowed us to describe the transformation of both maternal and fetal tissues, which in turn, results in the narrowing of the barrier between their bloodstreams. Unlike in other species, some specialized maternal cells (called decidual cells) were observed to be active until the end of gestation. These results suggest that, although there are similarities with other species, there are also characteristics specific to the development of the feline placenta. Many of our results could serve as the basis for further studies useful to understanding the normal role of the placenta in the nutrition and postnatal outcome of kittens, as well as alterations in the placenta.

## 1. Introduction

During gestation, domestic cats (*Felis catus*) develop a zonary girdle-shaped, chorioallantoic, and lamellar placenta at the equatorial area of the maternal–fetal units. Usually, there are hematophagous zones at the edge or in the middle of the zonary structure, but not so evident as in dogs. The chorion and the maternal tissues in those areas are widely separated by stagnant maternal blood [1]. The placental belt is constituted by the chorioallantoic membrane (CAM), the labyrinth, which is composed of a body and a front that is included in the maternal–fetal junction zone (JZ), and the glandular zone of the endometrium. The labyrinthine body is occupied by alternating fetal and maternal lamellae parallel to one another. Fetal lamellae comprise a vascularized mesenchymal axis lined by the trophoblast, while maternal lamellae contain only blood vessels and decidual cells (DCs), embedded in scarce connective tissue. Therefore, the maternal–fetal barrier is classified as endotheliochorial [2]. There are two trophoblast cell populations: cytotrophoblastic (CTB) discrete cells, and the syncytiotrophoblast (STB). CTB cells, through division, differentiation, and fusion, form the STB, which is in close contact with decidual cells; these cells differentiate from uterine stromal cells and become incorporated consistently into the labyrinth [1,3]. In the JZ, maternal and fetal tissues face each other.

Endotheliochorial placentas have been reviewed in several articles, such as Enders and Carter [4], Wooding and Burton [1], Miglino et al. [5], and Kowalewski et al. [6]. Particularly, the canine placenta has been addressed from different perspectives, e.g., Anderson [7] has described the ultrastructure of the placenta and fetal membranes between days 40 and 60 of gestation. Aralla et al. [8] have described the histological changes occurring from implantation to term in detail, and Sarli et al. [9] related histological findings and vascular density to puppies’ neonatal outcomes. Aspects such as vascularization [10], hormone production [11] and components of the extracellular matrix [12,13] have also been studied in this species. In addition, Diessler et al. [14] have reviewed decidual cells’ morphology and development, while Gualdoni et al. [15] have related matrix metalloproteinases-2 and -9 to trophoblastic invasiveness in dogs and cats.

Conversely, from the mid-twentieth century up to now, no more than 50 articles have been published specifically on the feline placenta, and only 12 of them address some morphological aspect. Malassiné described modifications of labyrinthine ultrastructure from day 45 of gestation [16], while Leiser portrayed the development of the trophoblast in early [17] and late [2] placentas. Most recent studies have focused on particular functional aspects, such as the regulation of amino acid transport [18], the production of growth factors and other signaling molecules [19,20,21], induction and post-transcriptional regulation of transport proteins [22,23], immunological and neuroendocrine regulators [24], and angiogenesis mediators [24,25,26]. The distribution of cytoskeletal proteins and the glycosylation pattern have been studied in mature placentas by Fernandez et al. [27], while Jones et al. [28] have described glycosylation dynamics throughout gestation, and Walter and Schonkypl [29] examined extracellular components and matrix-degrading enzymes. Also, Siemieniuch et al. [30,31], García Mitaceck et al. [32], and Fieni et al. [33] have contributed to knowledge in the field of feline pregnancy maintenance and interruption. Finally, Santos and Silva [34] have extensively reviewed molecular factors involved in the reproductive morphophysiology of the female domestic cat and included a rough overview of the placenta.

Even today, information about the histological features of each zone of the developing feline placenta remains scarce. A growing awareness of the normal morphology of this rapidly changing organ will help researchers and diagnostic pathologists to recognize placental lesions. For the reasons set out above, the objective of this work was to make a detailed description of the changes in the feline placenta during gestation.

## 2. Materials and Methods

### 2.1. Sampling and Macroscopic Procedures

Thirty-seven gestational sacs from pregnant crossbred queens were obtained by colleagues after owner-required and authorized ovariohysterectomies or cesareans; in most cases, the gestational age was unknown. The Institutional Committee for the Care and Use of Laboratory Animals (CICUAL, Facultad de Ciencias Veterinarias, Universidad Nacional de La Plata) approved all the procedures (protocols Nº 58-3-16T and 97-2-19T).

**Figure 1:**
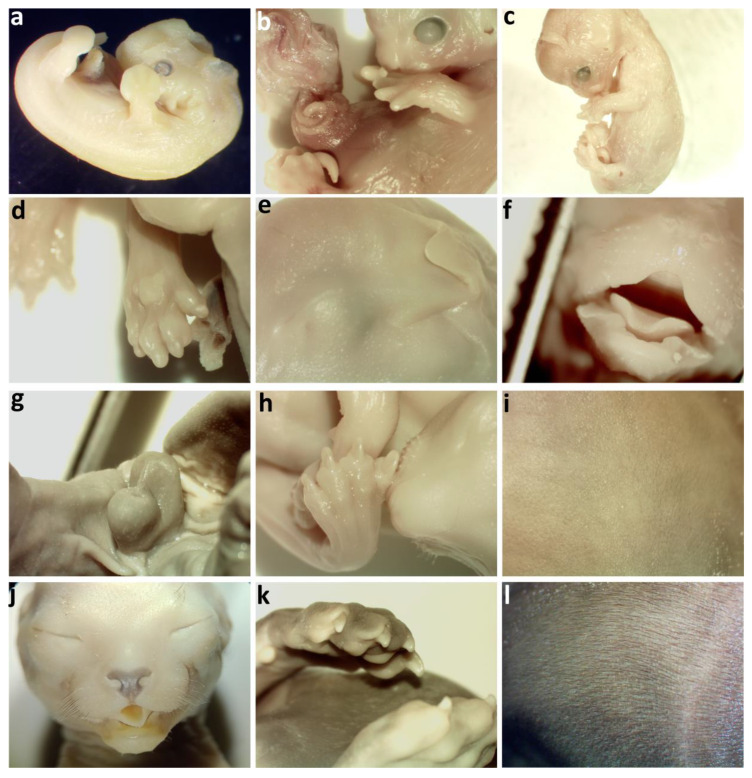
Sequence of embryos and fetuses showing some of the developmental features considered in this study. (**a**) Acoustic meatus forming, shallow grooves between forelimb digits, hind limb plate present; (**b**) forelimb digits separating distally, hind limb plate notched, tactile hair follicles on lips, snout, and above eyes; intestines herniated; (**c**) triangular pinna projecting rostrally, forelimb digits widely spread, hind limb digits separating distally; (**d**) plantar pads, claws forming; (**e**) eyelids fused, pinna covering acoustic meatus; (**f**) palate fused, tactile hairs present on the face; (**g**) visibly differentiated external genitalia; (**h**) claws hardening at the tips; (**i**) fine hairs appearing on the body; (**j**) nose pigmented, long tactile pigmented hairs on face; (**k**) claws white and hard; (**l**) fine hairs covering the body.

Embryos and fetuses were photographed, measured, and then examined under a Nikon SMZ 645 stereoscopic microscope coupled to an AmScope MU1000 digital camera connected to the image processing software AmScope v.3.7. Determination of the gestational age of each embryo or fetus was based on general external developmental characteristics, following the guidelines issued by Evans and Sack [35] and Knospe [36]. In addition to crown–rump length (CRL), the following characteristics were taken into consideration: formation and separation of the digits, shape and size of the pinna, presence of tactile hairs on the face, appearance of hair follicles and hair, hardening of the claws, and so forth (Figure 1).

**Figure 2:**
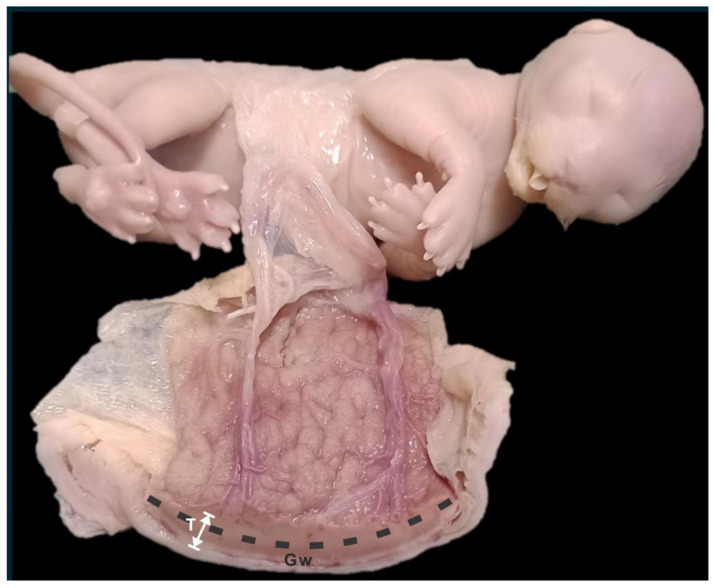
Fetus and feline placenta. Macroscopic sample. Gw: girdle width; T: placental thickness.

Macroscopic examination of either whole gravid uteri or isolated placentas was performed; placental girdle width was measured with a caliper (Figure 2). Each placenta was assigned to a gestational age according to the developmental stage of the corresponding embryo or fetus.

Samples up to 1 cm were collected from the girdle, fixed in 10% formalin for 24 h, then stored in 70° ethanol and processed using routine histological techniques. Sections of 3 µm were obtained and stained with hematoxylin and eosin (H&E). The PAS reaction and von Kossa (VK) silver nitrate method for calcium were also performed in selected samples.

In one case, additionally, fresh tissue from term placenta of four gestational sacs was available for ultrastructural analysis. These samples were conditioned and processed for high-resolution and transmission electron microscopy, following the protocols described by Cristofolini et al. [37].

### 2.2. Histological and Morphometrical Analyses

Images of the labyrinth and JZ from H&E-stained placental slides were captured using a digital camera (Leica ICC50W, Leica Microsystems, Wetzlar, Germany) mounted on a transmitted light microscope (Leica DM500,Leica Microsystems, Wetzlar, Germany) to carry out several measurements and achieve an in-depth description.

Initially, the thickness of the whole placenta, and the labyrinth in particular, was measured in each sample. The average lamellar width was obtained within the labyrinth after measuring nine lamellae from the intermediate zone (three per field, selecting three nonadjacent 20X fields throughout the labyrinth). Furthermore, the area occupied by decidual cells in the JZ and the labyrinth was calculated.

For the JZ, 100 DCs were manually measured in placentas from different gestational ages; their staining intensity was established, and the occupancy area was determined to perform the final counts. The percentage of DC occupation was set as the ratio of DC area: image area 40X*100:

[% DC occupancy = DC area/total area*100]

On the other hand, in the labyrinth, the counting of decidual cells present in the lamellae was performed manually, and the percentage of decidual cells was established based on the total area of the lamellae using the formula:

[% area occupied by decidual cells = decidual cell area/lamellar area]

All measurements were performed using the image analysis software ImagePro Plus, v6.3 (MediaCybernetics, Rockville, MD, USA).

After thorough observation and measurement, samples were clustered into five groups for histological description and morphometric analysis (A: 19–26 dpc; B: 27–38 dpc; C: 39–48 dpc; D: 49–59 dpc; E: 60–term).

### 2.3. Statistical Analysis

Variables such as the placental width and thickness, lamellar width, percentage of decidual cells/total area in the junctional zone, and percentage of decidual cells/lamellar area were analyzed using InfoStat software Version 2019e 2019e by one-way ANOVA followed by the Tukey test to determine intergroup differences; *p*-value < 0.05 was considered statistically significant.

In addition, a correlation coefficient was calculated to determine how, and to what extent, pairs of variables, e.g., placental thickness and gestational age, were associated. For both positive and negative correlations, <0.5 was considered low, 0.5 ≥ 0.7 as medium, 0.7 ≥ 0.9 as high, and only >0.9 as very high. To model a linear trend observed by scatterplots, a regression line is shown. The distance between each datum and the mean value (e.g., the width of the placenta at each gestational age in days vs. the lamellar width means) is displayed. This model explains the proportion of data variation around the mean value (see figures in the “Results” section).

## 3. Results

### 3.1. Gestational Age

**Figure 3:**
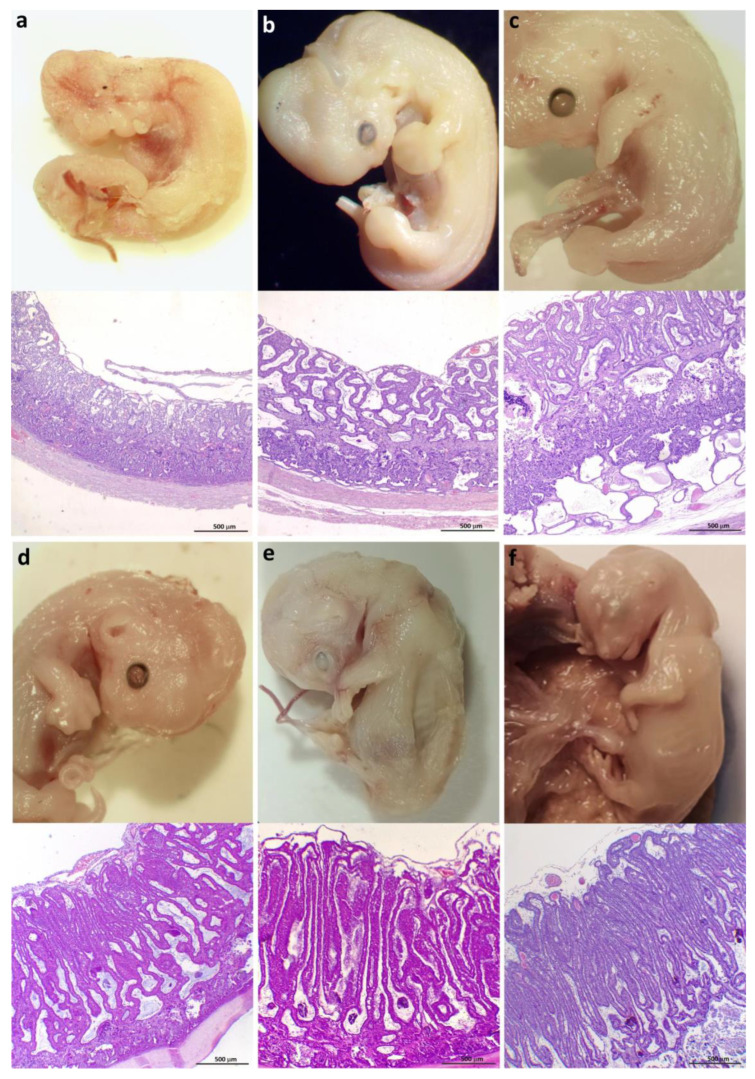
Feline embryos (**a**–**e**) and fetus (**f**) and their corresponding placental samples: (**a**) 19 dpc; (**b**) 21 dpc; (**c**) 22 dpc; (**d**) 23 dpc; (**e**) 27 dpc; (**f**) 31 dpc. H&E.

**Figure 4:**
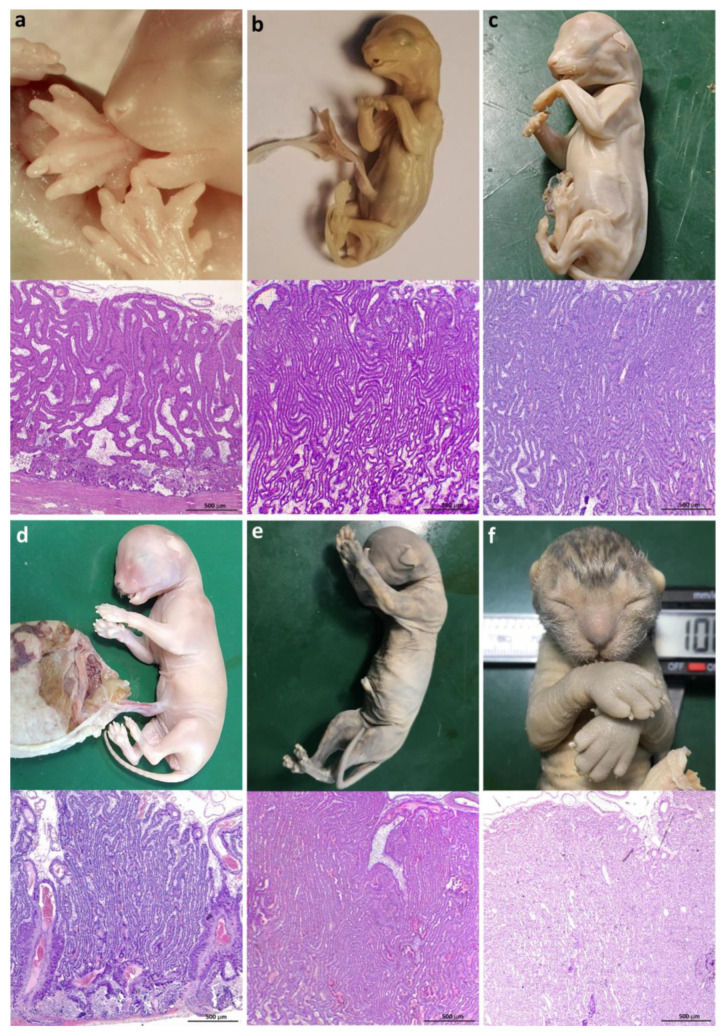
Feline fetuses and their corresponding placental samples: (**a**) 34 dpc; (**b**) 38 dpc; (**c**) 40 dpc; (**d**) 46 dpc; (**e**) 50 dpc; (**f**) 56 dpc. H&E.

Based on developmental features and considering the proposed grouping, the embryos and fetuses under study were distributed as follows: eleven cases (19–26 days post-coitum -dpc-), eight cases (27–38 dpc), nine cases (39–48 dpc), five cases (49–59 dpc), and four cases (≥60 dpc). Their placentas have been, therefore, described under these labels (Figure 3 and Figure 4).

### 3.2. Histological Features of Feline Placentas in Different Stages of Development

*Group A: 19–26 days*

**Figure 5:**
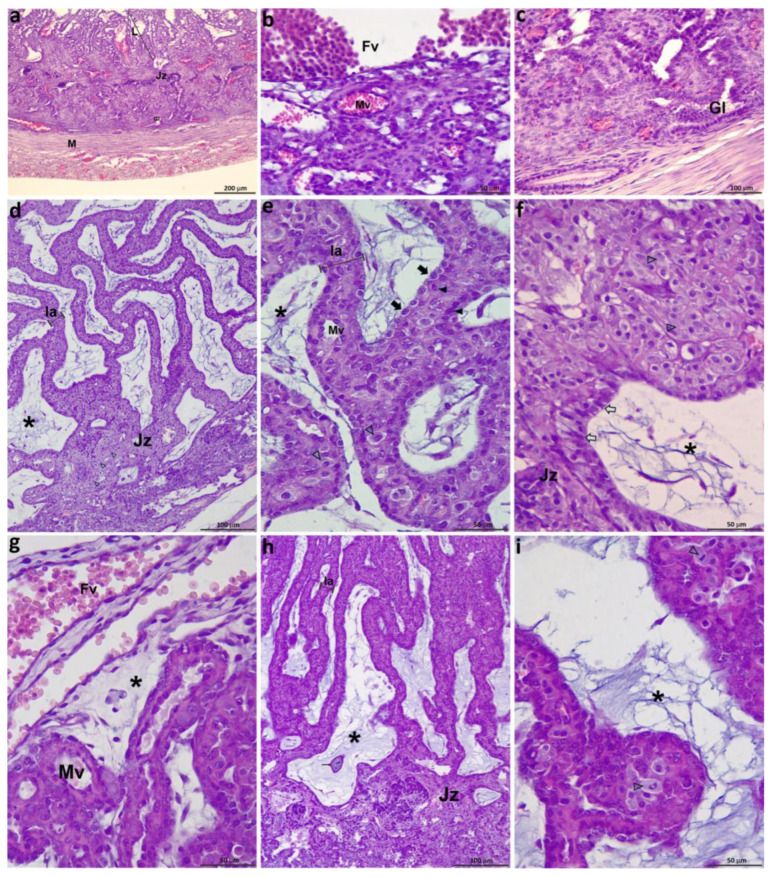
Feline placentas from group A: (**a**–**c**): 19 dpc.; (**d**–**f**) 21 dpc; (**g**–**i**) 23 dpc. (**a**) Labyrinth not fully developed yet, wide junctional zone, myometrium; (**b**) fetal and maternal vessels in the fetal side; (**c**) maternal glands; (**d**) labyrinth and junctional zone; (**e**) labyrinth; (**f**) decidual cells and villous tip in the JZ from (**d**); (**g**) fetal vessels in the CAM; (**h**) labyrinth and junctional zone; (**i**) mitotic figures in decidual cells. Fv: fetal vessels; Gl: uterine glands; Jz: junctional zone; L: incipient labyrinth; la: lamella; M: myometrium; Mv: maternal vessels; asterisks: mesenchyme; full arrowheads: STB; empty arrowheads: DCs; empty arrows: Jz trophoblast; thick full arrows: CTB; thin arrows: calcium. Each scale bar has been labeled. H&E.

In samples from day 19, an incipient labyrinth was already observed, although the lamellae were not yet fully developed (Figure 3a and Figure 5a). Regarding the fetal side and the adjacent portion of the labyrinth, the villi mesenchyme was scarce, and trophoblast populations were not differentiated. Most fetal vessels in the CAM were venules and contained nucleated erythrocytes. Some maternal vessels beside the CAM had a cuboidal epithelium (Figure 5b).

In the JZ, the columnar trophoblast of the villi tips was observed close to the endometrium. DCs could not be observed yet; endometrial glands had a cubic or cylindrical epithelium, and their lumen was often full of secretions and detritus (Figure 5c).

On days 20 and 21, within fetal vessels of the CAM, cells from the erythroid lineage were largely still nucleated. Several labyrinthine lamellae had already formed; they were thick, convoluted, and clearly delimited by an abundant mesenchyme (Figure 3b and Figure 5d). Trophoblast differentiation into CTB cells and STB had begun. The CTB cells were cubic with a pale cytoplasm and had a spherical, loose nucleus; they formed a continuous layer covering the lamella. In contrast, the STB nuclei were oval and dense. Maternal vessels within the labyrinthine lamellae had a thin basal membrane and a thick endothelium, and there were some small decidual cells (Figure 5e). In samples from day 20 onwards, a positive basophilic granular substance compatible with calcium, positive after the von Kossa method, could be observed in mesenchymal blood vessels.

In the JZ, decidual cells were abundant and grouped, forming compact areas. They were similar to those in the lamellae. Villi tips were wide and mainly covered by CTB cells and STB, although denuded of STB in some areas (Figure 5f).

Between 22 and 26 days, the ratio of nucleated to enucleated red blood cells began to decrease (Figure 5g). The labyrinth was thicker. The lamellae were arranged more parallel than in samples from previous days and were wider towards the third of the labyrinthine body nearest to the CAM (Figure 3c). The mesenchyme was abundant and bluish, with a mucinous aspect; some capillaries and small venules were observed there (Figure 3d and Figure 5h). Labyrinthine DCs were abundant, small, and had pale cytoplasm; they were arranged in groups. Numerous mitotic figures could be observed in CTB and decidual cells (Figure 5i). The JZ appeared wider, and abundant detritus was present. Also, clusters of small DCs were observed (Figure 5h).

*Group B: 27–38 days*

**Figure 6:**
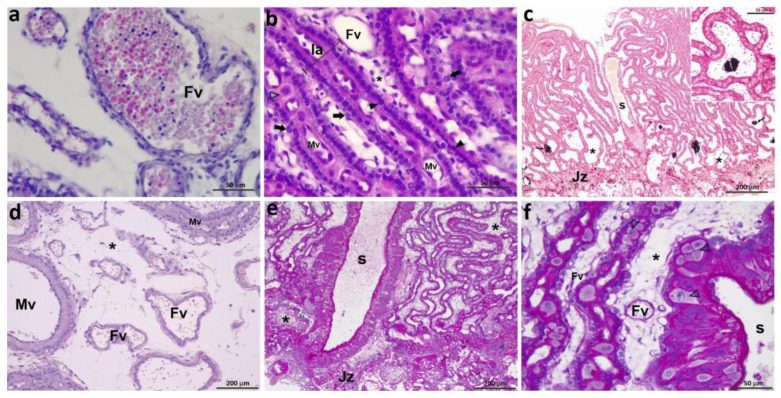
Feline placentas from groups B and C: (**a**) 31 dpc.; (**b**,**c**) 35 dpc; (**d**) 39 dpc; (**e**,**f**) 45 dpc. (**a**) Fetal vessels from the CAM; (**b**) labyrinth; (**c**) labyrinth, JZ, stem artery, and calcium within vessels. Inset: detail of calcium; (**d**) fetal vessels from the CAM; (**e**,**f**) lamellae and stem artery. Fv: fetal vessels; Jz: junctional zone; la: lamella; Mv: maternal vessels; S: stem artery; asterisks: mesenchyme, empty arrowheads: DCs; full arrowheads: STB; thick arrows: CTB; thin arrows: calcium. Each scale bar has been labeled. (**a**,**b**,**d**) H&E, (**c**) VK method, (**e**,**f**) PAS reaction.

The labyrinth appeared elongated, and lamellae were more densely packed than in previous stages (Figure 3e,f and Figure 4a,b). The vessels in the CAM had become numerous and larger, and veins and arteries were observed. Very few fetal erythrocytes retained the nucleus (Figure 6a). The number of lamellae was higher in these samples, and they were already parallel in many sectors. Syncytial nuclei, increasingly abundant, were ordered in rows (Figure 6b). Anisocytotic DCs could be observed in the lamellae, and maternal endothelia remained cuboidal. Mesenchyme was scarcer than in samples from earlier placentas; the positive von Kossa granular substance could still be observed. Maternal stem arteries became apparent in these samples (Figure 6c). The JZ was narrower than in previous stages. In some villi tips, the CTB is denuded of STB. Clusters of small decidual cells are also observed in this area.

*Group C: 39–48 days*

The labyrinth became more compacted; their lamellae were narrower and parallel, although they presented some bends (Figure 4c,d). The fetal red blood cells were already anucleate (Figure 6d). One or two large DCs were found across the lamellae. Their nuclei had loose chromatin, and several of them were binucleate. STB cytoplasm began to look vacuolated. Villi tips were broader and shorter; some were covered only by CTB. Clusters of small decidual cells could still be observed in the JZ. The stem arteries were remarkably large and surrounded by STB, the nuclei of which were radially arranged (Figure 6e,f).

*Group D: 49–59 days*

**Figure 7:**
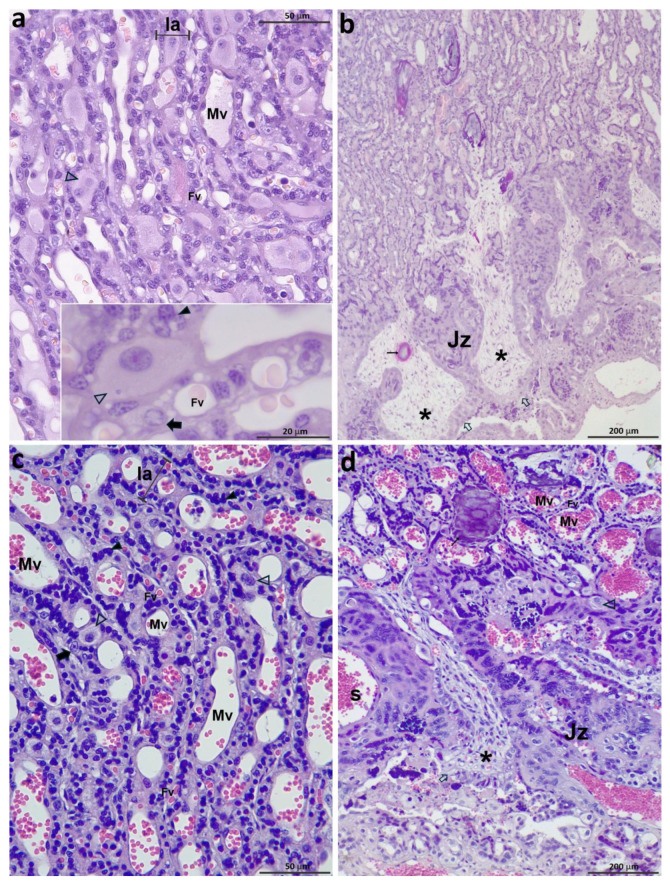
Feline placentas from groups D and E: (**a**,**b**) 56 dpc; (**c**,**d**) 62 dpc. (**a**) Lamellae; (**b**) labyrinth and JZ; (**c**) lamellae; (**d**) labyrinth and JZ. Fv: fetal vessels; Jz: junctional zone; la: lamella; Mv: maternal vessels; S: stem artery; asterisks: fetal connective tissue; empty arrows: Jz trophoblast; empty arrowheads: DCs; full arrowheads: STB; thick full arrows: CTB; thin arrows: calcium. Each scale bar has been labeled. H&E.

In the CAM, large blood vessels had developed. A high density of parallel lamellae per area, with scant mesenchyme among them, resulted in the compact labyrinth typical of a mature placenta (Figure 4e,f). Isolated CTB cells could be seen. The syncytiotrophoblast had abundant vacuoles, and the chromatin was arranged in clumps, in contrast to what had been observed in earlier stages. At the end of the stage, small fetal capillaries were observed interspersed with trophoblastic cells (Figure 7a). Decidual cells were large, and each one occupied the width of a lamella. The maternal vessels had flat endothelia except for the labyrinth zone next to the CAM, where they appeared higher. Uncovered CTB was still observed in some villi tips. The mesenchymal matrix resembled mature connective tissue (Figure 7b).

*Group E: 60 days to term*

The labyrinth was very dense, and the lamellae were narrow and extremely close to each other (Figure 7c). Many fetal arterioles, venules, and capillaries were in the mesenchyme between the lamellae. Cytotrophoblastic cells were extremely scarce, and the syncytiotrophoblast was flat and intensely stained. The maternal and fetal vessels were thin-walled. The distance between maternal and fetal blood vessels was minimal, frequently making it quite challenging to discern between the components of one individual and the other in this mixed organ. The decidual cells were scant, giant, and isolated (Figure 7d).

To summarize the histological findings, lamellae became increasingly abundant, long, parallel, and narrow, while the mesenchymal villous core, which had been abundant and predominantly mucinous, progressively became scarcer, and capillaries became increasingly numerous. Calcium was observed in samples from 20 dpc to term. CTB cells, initially abundant, proliferative and comprising a complete layer, gradually incorporated into the STB; only a few of them remained isolated in situ and discernible towards term. Accordingly, larger quantities of STB nuclei were observed. STB cytoplasm was conspicuously vacuolated and progressively reduced. Regarding the labyrinthine maternal components, endothelial height decreased from the vascular segments next to the CAM to those nearer to the uterus, as it also did from early to late pregnancy. The placental barrier became thinner. Decidual cells gradually incorporated into the lamellae from the compact masses they had formed in the JZ, becoming less abundant and larger. At the JZ, which tended to thin out, villi tips were covered almost entirely in CTB cells, and their axis was formed by increasingly mature connective tissue.

### 3.3. Ultrastructural Analysis

**Figure 8:**
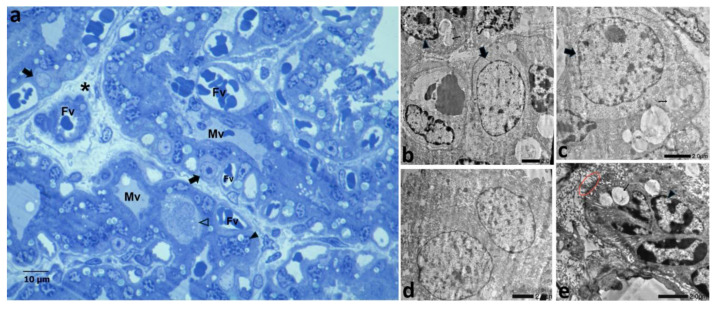
Feline placenta, 60 dpc: (**a**) placental lamellae in a semithin section; (**b**–**e**) ultrastructure of a CTB cell, STB, and a fetal vessel. (**a**) Lamellae; (**b**) CTB and STB; (**c**) CTB; (**d**) binucleated decidual cell; (**e**) STB. Fv: fetal vessels; Mv: maternal vessels; asterisks: fetal connective tissue; empty arrowheads: DCs; full arrowheads: STB; red circle: microvilli; thick arrows: CTB; thin arrows: STB vesicles. Each scale bar has been labeled. (**a**) High-resolution optical microscopy, (**b**–**e**) TEM.

In the villus core, mesenchymal cells with typical, irregular and indented nuclei were seen; sections of collagen fibrils were also found. Fetal endothelial cells exhibited abundant smooth membrane invaginations of 70–79 µm, resembling caveolae, numerous polysomes, and scant rough-reticulum cisternae. Light cytoplasm on CTB cells was highlighted by the dark electron-dense syncytiotrophoblast cell layer. The former had euchromatic nuclei with an evident nucleolus and were linked to the STB by desmosomes. The STB surface was increased by means of irregular protrusions and folding. The dark cytoplasm showed a well-developed rough reticulum and lipidic inclusions. Nuclei in the syncytium had a typical spotted appearance due to clumped chromatin. Mono and binucleated large decidual cells had irregularly dense cytoplasm, lighter in the cell cortex, with abundant and heterogeneous vesicles and rough-reticulum cisternae (Figure 8).

### 3.4. Morphometric Analysis

As expected, the length of the embryo fetuses increases constantly during gestation (from 10.75 up to 129 mm), growing markedly after 31 days, already in the fetal stages.

**Figure 9:**
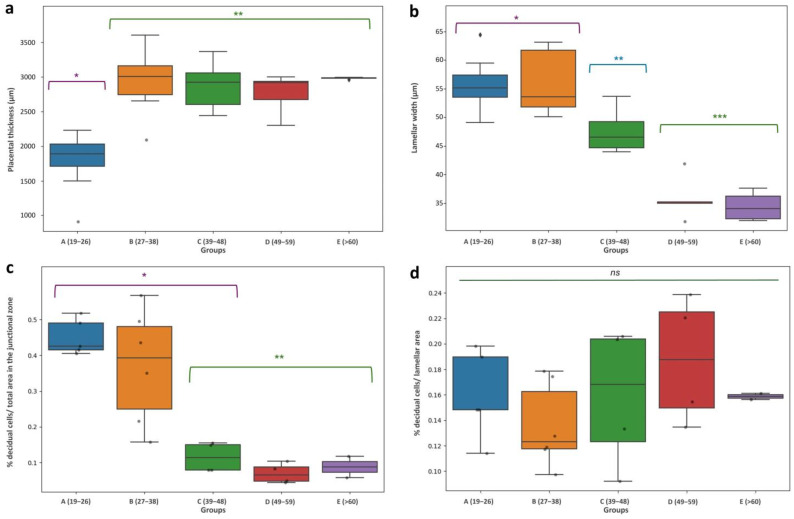
Morphometrical and statistical analyses. Intergroup differences. (**a**) Placental thickness; (**b**) lamellar width; (**c**) percentage of decidual cells/total area in the JZ; (**d**) percentage of decidual cells/lamellar area. The Box-and-whisker plot illustrates the spread of the data for each group. Different symbols above bars represent significant differences between groups, *ns*: no significant differences. *, **, ***: “Different symbols above bars represent significant differences between groups.

The placental girdle width varied in a range between 18.4 and 41.6 mm, and there were no significant differences between groups during pregnancy. Concerning placental thickness, it ranged from 1495 to 3360 µm, and a very slight decrease was observed after 39 dpc. There were significant differences between group A (19–26 dpc) and the rest, among which no significant differences were found (Figure 9a).

With respect to the labyrinth, the average width of the lamellae varied from 64.4 to 31.8 µm, decreasing progressively during gestation. Differences between groups A and B did not meet statistical significance, whereas differences between them and groups C, D, and E were significant. In addition, between the D and E groups, there were no significant differences either (Figure 9b).

Regarding the relative decidual area (decidual area/total area in a particular zone), while it decreased dramatically in JZ as gestation progressed, especially after 38 dpc (Figure 9c), no significant statistical differences were obtained when comparing the area of lamellar decidual cells (Figure 9d).

**Figure 10:**
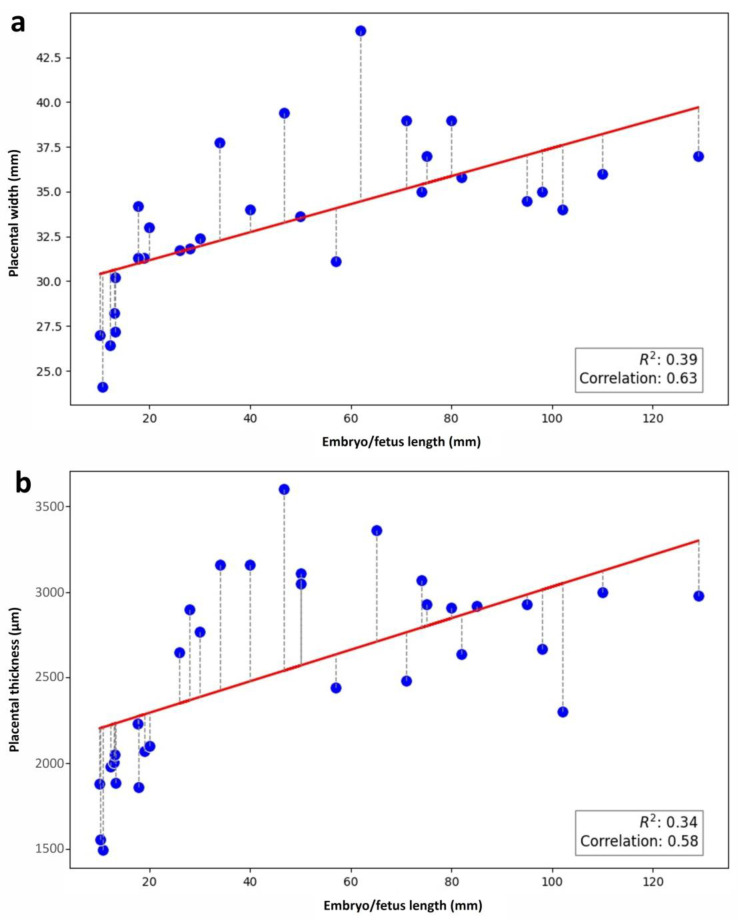
Morphometrical and statistical analyses. Correlations: (**a**) embryo/fetus length vs. placental girdle width; and (**b**) embryo/fetus length vs. placental thickness. Each dot represents one sample.

A positive medium correlation was found between the length of the fetuses and both the width and thickness of their placental girdle: r = 0.63 and r = 0.58, respectively (Figure 10).

**Figure 11:**
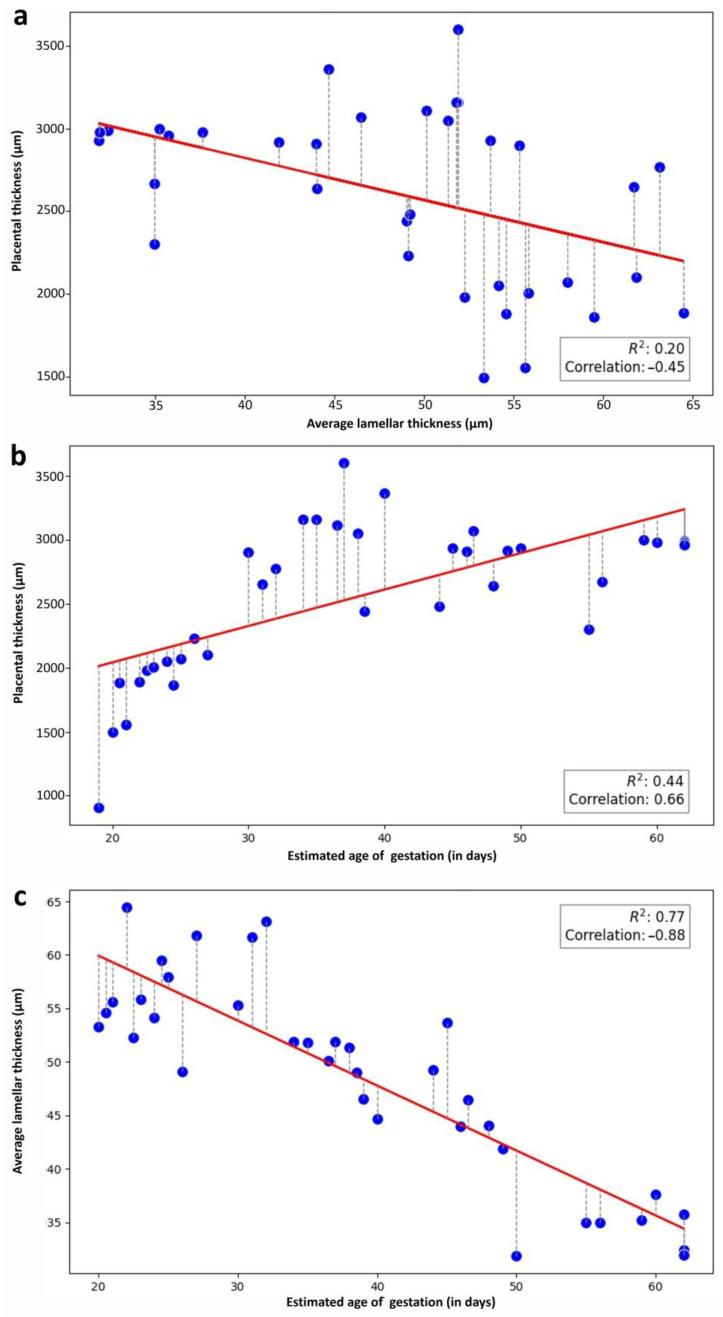
Morphometrical and statistical analyses. Correlations: (**a**) placental thickness and average lamellar thickness, (**b**) placental thickness and estimated age of gestation, and (**c**) average lamellar thickness and estimated age of gestation. In subfigures (**a**,**b**), each dot represents a sample, while in (**c**) the dots represent the mean measurements of lamellae thickness.

In contrast, a negative low correlation was found between placental thickness and lamellar width (r = −0.45) (Figure 11a). Placental thickness was higher in later placentas than in earlier ones (r = 0.66) (Figure 11b), while lamellar width decreased towards term (the mean decreased from 55 µm in group A to 34 µm in group E). This last comparison showed a strong correlation (r = −0.88) (Figure 11c).

## 4. Discussion

This work intends to provide further detail about the placental organogenesis in cats, and particularly to fill in the gaps detected regarding placentas from 25–30 dpc to 60 dpc, a period which has been relatively under-examined in the reviewed articles. Additionally, given our lack of data on the mating date of the queens (which might well be the case in an abortion diagnosis scenario), gestational age was estimated by several features, while most of the other authors took into account only (or mostly) direct or indirect fetal measurements [24,26,38,39,40,41]. Qualitative, developmental features, rather than those more related to growth, such as weight or length, might be less affected by maternal and gestational parameters such as queen’s age, weight, breed, and litter size, which were shown to impact reproductive parameters as gestation length [42,43].

### 4.1. Fetal Length and Placental Macroscopic Findings

A rapid increase in fetal growth from 31 dpc was noted in this study, virtually in agreement with Knospe [36], who reported that rise as beginning at 32 dpc. Our data regarding the length of embryos and fetuses partially agree with those from other authors, who reported the embryo/fetal length of 5–10 individuals [38,39]; one of the reports lacked data for >45 dpc gestations [39]. The greater difference was found for 20 dpc embryos: here, the CRL was 10.15 mm, while it was reported as 15 mm by Illanes et al. [38], and as 7.6 mm by Illanes et al. [39]. These differences might be due to the methods employed: Julio Illanes et al. first measured the length and weight of the embryos and fetuses and only then estimated their gestational age and described developmental features [38]; in Illanes et al.’s work [39], on the other hand, they were already aware of gestational age, but measurements were performed by ultrasonography. In addition, only Persian and Thai pregnant queens were included in that study. Our data regarding fetal length at the latest stage of gestation (≥60 dpc) matched those reported by Illanes [38].

With regard to placental girdle width, our data corresponded to results reported in the articles cited above. As these studies lack information for some gestational intervals, and also regarding embryo/fetal length, it was not possible to perform comparisons of placental width variation throughout pregnancy.

### 4.2. Placental Thickness

Placental thickness has also been estimated—or at least illustrated—in several other works. Here, a quite regular increment was observed from 1.4 mm in 19 dpc samples to more than double that thickness at 39 dpc, when it peaked and reached a plateau; then, only a slight decrease was recorded. The peak of such striking and abrupt placental thickening has been reported to occur at 34, 36, or 38 dpc, according to other research groups [26,38,39]. A slight post-peak decrease was also observed in G-Basso et al.’s work [26]; conversely, the remaining two groups reported thickening, although minimal, towards term [38,39]. Given these data, plus some information that could be obtained from the reported figures, there seems to be a consensus that a higher placental growth rate takes place during the second third of gestation [5,26,29,38,39]. Both Santos and Silva [34] and Wooding and Burton [1] illustrated placentas from widely separated gestational ages; it is clearly shown that the depicted 60 dpc placentas were at least double the thickness of the 25–30 dpc ones, but they did not contribute images from any intermediate stage of placentation.

### 4.3. Labyrinthine Development

Changes in labyrinthine number, arrangement, length, and parallelism largely depend on vascular development and trophoblast proliferation, while the general appearance of the labyrinth and lamellar density also result from changes in the villi mesenchymal core.

Lamellar width progressively decreased (from 64.4 to 31.8 μm); this finding, which entails the thinning of the placental hemotrophic barrier, agrees with findings reported by several authors [1,16,17,26].

The main processes related to trophoblastic differentiation and turnover have been characterized in species developing a syncytium, including cats [1,2,17,44]. CTB cells are in a proliferative state and initially constitute a complete layer anchored to a basal lamina; this was the case in placentas from groups A and B, with full or almost complete CTB layers. As gestation progresses, proliferation declines and fusion of individual cells to the STB increases [45], which matches the detection of incomplete layers, composed of short 3–6 cell rows in samples from 39–48 dpc, and rows of 2–3 cells in those from group D, as well as the finding of scarce and isolated CTB cells in samples around 60 dpc. Accordingly, the number of syncytial nuclei increases and they are arranged in a single row. Factors affecting the dynamics of proliferation, fusion (and apoptosis) of CTB cells, as well as their timing, constitute an interesting topic still unexplored in feline placentas. It is known that villous mesenchyme produces CTB regulatory signals, so changes in this tissue affect the CTB proliferation rate and survival [46]. The shrinking of the mesenchymal villous core at around 38–39 days found here (noticeable even in low magnification figures) went hand in hand with the loss of CTB layer continuity. Few apoptotic nuclei can be detected at this stage (Gomez Castro et al., unpublished data).

The key mechanisms by which STB integrity is maintained involve termination of the CTB cells’ capability for self-renewal and upregulation of genes needed for differentiation and fusion into the overlying syncytium [reviewed in [45]]; many substances are involved in these mechanisms, with syncytins playing a major role [47]. In this respect, although this process has not yet been studied in cats, it has been reported that the fusogenic retroviral gene *syncitin-Car1* is conserved in *Felis catus,* with placental-specific expression [48]. One of its regulators, galectin-1, has been detected in feline CTB cells, being more abundant in placentas from 40 days [20], when increasingly abundant STB nuclei are observed. Aging of the STB, on the other hand, has been studied, especially in human hemochorial placentas, in which this process results in the shedding of syncytial knots directly into maternal blood. The release of the knots is regarded as the end stage of apoptosis, being common only after 32 weeks of gestation in humans [49,50]. The fusion of CTB cells to the STB would not, on its own, explain the lamellar thinning described above, as unless another process occurred, the total trophoblast volume would remain unchanged. Further studies, particularly regarding syncytial apoptosis, are necessary to comprehensively understand the trophoblast development in the feline placenta.

The structure and ultrastructure of CTB cells and STB corresponded to previous descriptions [1,2,16]. A vacuolated appearance, as a result of lipid droplet accumulation, was observed in the STB, and to a far lesser extent, in CTB cells, mainly in placentas from groups C-E (≥39 dpc). It has been described in early placentas by Wislocki and Dempsey [51], who studied both early and late placentas and described these droplets as syncytial but also appreciable in CTB cells. Malassiné, who studied feline placentas from 45 dpc onwards, named lipid droplets as “liposomes” and reported that syncytial ones were bigger and far more abundant [16]. The fetal component facing maternal vessels in the feline placenta is the STB; its central role is maternal–fetal and fetal–maternal transfer, including the transfer of lipids [52]. It is the site of the highest metabolic activity in the placenta, and besides transport, catabolism and resynthesis of lipids also occur [44]. Once in the trophoblast cytosol, non-esterified fatty acids can bind to proteins for transfer to the fetal circulation, be oxidized or re-esterified, and stored as triglycerides in lipid droplets [53]. Although the main trophoblastic lipid storage is syncytial, it also takes place in CTB cells (e.g., it was reported that incorporation of long-chain fatty acids is higher in CTB cells rather than, as expected, in the outer STB layer [54,55]. As the transfer of fatty acids across the placenta largely follows a maternofetal concentration gradient, its rate depends on the maternal lipid profile throughout gestation. In humans, it is known that fatty acids are mobilized from maternal lipid stores, especially in the last trimester of gestation [56,57]. Although, as far as we are aware, placental lipid transport has not been studied in cats, it seems evident from the histological results that trophoblastic storage of lipids is mainly syncytial and remarkably greater in feline placentas during the last third of pregnancy.

Sarli et al., who found placental calcification in canine placentas obtained by cesarean section, classified this process into three types: focal, linear, and sclerotic. The former was not associated with degeneration, necrosis, and/or inflammation, was found in the 55% of placentas from healthy pregnancies, and was considered as physiological. They located the basophilic granular material in trophoblastic lamellae, without specifying the particular structure [9]. While they mentioned that this is a feature of various species and that it could be interpreted as a background finding resulting from fetal blood pH, based on reports by Haschek et al. [58], we could not find any other reference to this phenomenon. Walter and Schönkypl [29] mentioned a finding of calcification, regarding it as “common” and locating it within the villi tips of the mesenchyme, in feline placentas from 40 days on. In a preliminary study, Denti et. al. reported placental mineralization in cats; it was restricted to 15% of the samples and consisted of lamellar multiple foci; data regarding the gestational age of the “positive” cases were not stated [59]. Here, calcium deposition was observed within fetal vessels, predominantly at villi tips, and regularly throughout gestation, including in term placentas; therefore, a relation to specific developmental events of the locomotor system seems unlikely.

Our finding that the endothelia of maternal vessels on the lamellar axis (particularly those closest to the CAM) exhibit a cuboidal shape until after 45 dpc, and then begin to flatten, almost matches the observations of Jones et al. [28], who reported that maternal vessels are lined with plump endothelial cells, which appear thinner around 44 dpc. G-Basso et al. [26] mentioned that the endothelium undergoes thinning, whereas Walter and S. [29] described cuboidal endothelia in placentas until 40 dpc but omitted further descriptions. Neither study provides specific details on the timing or mechanisms underlying these changes in endothelial cells.

The heterogeneity of endothelial cells across different types of blood vessels is evident with regard to morphological aspects, such as cell height, as well as the molecular and functional levels. High endothelium venules are characteristically found in lymph nodes, where they are involved in lymphocyte homing by adhesion and migration through the vessel walls [60]. In this location, the height of the endothelial cells depends on lymph flow, lymphocyte influx and, presumably, on up/downregulation of membrane glycans [61]. Whether endothelial cell plumpness in placental maternal vessels depends on flow pressure or on certain signals, or constitutes a particular stage of angiogenesis mediated by paracrine factors could be an interesting issue to study. Unlike other above-discussed research topics—regarding which at least hemochorial placenta data are available—the issue of labyrinthine maternal vessel development has been hardly studied. This may be simply due to the fact that the hemochorial placental barrier, typical of primates and most experimental animals, lacks maternal vessels.

In regard to the reduction in the placental barrier, Malassiné [16] reported that it diminishes from 15 to 2 µm from 45 dpc to 63 dpc, while Leiser and Koob [2] mentioned, as we found, that the barrier thins out until it reaches 1.5 µm. Besides the thinning of the placental barrier, lamellar growth and lengthening—with a consequent increase in the exchange area of the interhemal membrane—also favors solute transport; consequently, fetal requirements may be fulfilled [1,62]. As well as these morphological features, it was also emphasized that placental efficiency (defined as grams of fetus per gram of placenta) largely relies upon the abundance, activity, and localization of specific transporters, crucial for the transfer of essential nutrients for fetal growth, in the placental membranes [63]. While the molecular exchange across the placental barrier has been extensively studied in humans and other species with hemochorial placentas, the endotheliochorial placental barrier in domestic carnivores remains poorly understood. There are limited data on transporters, nutrient transport indices, and placental efficiency. A detailed investigation into placental cells activity is essential for understanding the physiological processes in this highly dynamic organ, which, as Fowden [63] suggests, adapts to both environmental conditions and the fetal organism’s needs.

### 4.4. Decidual Cells

Unlike those species in which decidual tissue gradually thins out and ultimately disappears, a feature that is regarded as the basal state for eutherian decidualization [64], in cats, decidualization lasts from 20 dpc to term, as was demonstrated in this work, in agreement with Boomsma’s findings [19]. In the JZ, large quantities of small, pale, and mitotically active cells form a compact plaque between the labyrinth and the glandular zone, as noted in our preliminary report [65]. Its relative area decreases dramatically during the second half of pregnancy, as the differentiating DCs become progressively integrated into the labyrinthine body, sandwiched between rapidly expanding chorionic villi.

We found that lamellar decidual cells became larger in early to late placentas, although less abundant; therefore, the relative decidual area remained without significant changes in that region. Many of the bigger DCs were binucleated (or, only exceptionally, tri- or tetranucleated), as opposed to junctional DCs, which were consistently mononucleated. Multinucleation constitutes a hallmark of terminal differentiation in DCs, as well as in cardiomyocytes, osteoclasts, and other cell types [66,67]. The development of such large cells, sometimes designated as “giant” cells, largely depends on the increase in the total DNA content, which allows for the override of the usual cell size controls [68]. Decidual cell size might have a great impact on placental biology, as the development of tissues containing few, large polyploid cells, especially in rapidly developing organs, is metabolically advantageous when compared to tissues containing numerous but small diploid cells [68]. Feline DCs are known to express a variety of substances, such as insulin-like growth factor-binding protein A, prolactin [69,70], relaxin [71], orexin, leptin [72,73], and galectins [20], among others. A potential differential biosynthetic capacity between moderately and terminally differentiated lamellar DCs in the feline species constitutes an interesting topic for future studies.

Regarding the binucleated condition observed, it might have been a result of the failure in the cytokinetic process, a subtype of endomitosis that is also reported in other physiological states [74]. As neither spindle elongation nor incipient cleavage furrows were seen, this failure might have occurred early. In addition, taking into account that a decrease in DC number was determined, binucleation may have also been a result of cell fusion, a process that also coexists with incomplete cytokinesis as a mechanism of polyploidization in other cell types [67]. Apoptosis of DCs might also account for the number decrease, although apoptotic decidual cells are minimally detected, at least in mice [reviewed in [66]].

Mononucleated DCs, most probably those of intermediate size and higher nuclear–cytoplasmic ratio, might also be polyploid in the feline placenta, but that status was not evaluated in this work. If that were the case, they could have undergone other mechanisms of polyploidy, such as truncated karyokinesis or an endoreplicative cycle (a switch from the normal G1–S–G2–M cycle to an S–G cycle), with rounds of DNA replication without cell division [75]. Dysregulation of CdKs and cyclins, reported in endoreplicative decidual cells, has not been studied yet in carnivoran species.

## 5. Conclusions

This study provides a detailed description of placental development in domestic cats, emphasizing the period from 30 to 60 dpc, which has been relatively unexplored previously. Changes in lamellar structure and dimensions ultimately lead to the thinning of the interhemal barrier, which is necessary to meet fetal demands for development and growth. The precise description of these temporal modifications, as reported here, may be helpful to complement functional studies.

In addition, the role of the syncytium in solute transport has been studied only to some extent in cats, and STB also has less explored functions concerning nutrient metabolism and synthesis of signaling molecules. This report of a progressive rise in syncytial nuclei number, along with a reduction in STB volume, raises several questions related, for instance, to its transcriptional activity during pregnancy.

Furthermore, the persistence of decidual cells in the feline placenta until term, as a derived feature, as well as data about their location, phenotype, and number throughout pregnancy, are relevant to attain deeper insights into these cells’ dynamics and function.

The detection of calcium throughout gestation was a striking finding, leaving space for several interpretations, as it might or might not be meaningful for fetal development. It is somewhat surprising that it was reported only in one article describing mature placentas; however, forthcoming analyses may shed light on this issue.

Besides constituting a detailed description of feline placental morphogenesis, our findings might be valuable for the elucidation of mechanisms behind trophoblast biology and vascular development. Their relationship to placental efficiency is an open field of research, relevant to both comparative placentology and studies on applied cat reproductive biology.

## Data Availability

Data regarding measurements are available from the corresponding authors, G.G.C. and C.G.B., upon reasonable request.

## Abbreviations

The following abbreviations are used in this manuscript:

CAM	Chorioallantoic membrane
JZ	Junction zone
DC	Decidual cell
CTB	Cytotrophoblastic discrete cell
STB	Syncytiotrophoblast
H&E	Hematoxylin and eosin
VK	von Kossa
CRL	Crown–rump length
